# Psychological Outcomes and Research Engagement Among PhD Students in Medicine and Health Sciences: A Systematic Review

**DOI:** 10.3390/bs16091575

**Published:** 2026-09-04

**Authors:** Lea Dumic, Nika Lovrincevic Pavlovic, Maja Miskulin

**Affiliations:** Faculty of Medicine Osijek, Josip Juraj Strossmayer University of Osijek, 31000 Osijek, Croatia; ldumic@mefos.hr (L.D.); maja.miskulin@mefos.hr (M.M.)

**Keywords:** education, graduate, medicine, biomedical research, burnout, impostor syndrome, wellbeing, mental health, psychological distress, motivation

## Abstract

Background: In recent decades, doctoral education has undergone a profound transformation. As a result, PhD students have become an increasingly vulnerable population, facing greater risk of mental health problems than the general population. A particularly important subgroup comprises PhD students in medicine and health sciences, who often combine doctoral training with clinical work. Their dual roles expose them to additional pressures, with an increased risk of burnout. Given that their context differs considerably from the typical university environment, in this systematic review, we aim to synthesize the current evidence on burnout, impostor syndrome, and research engagement, along with related indicators of psychological distress, among PhD students in medicine and health sciences, as well as identify key risk and protective factors and highlight gaps for future research. Methods: A systematic literature search was conducted in March 2026 across the Web of Science, Scopus, and Ovid MEDLINE databases. Following a screening process based on PRISMA guidelines, 27 studies were included; due to substantial heterogeneity, findings were synthesized narratively. Results: Psychological difficulties appeared to be more strongly associated with institutional factors rather than personal traits. Burnout was highly prevalent and linked to professional isolation and poor relationships with supervisors. Impostor syndrome emerged as a recurrent theme in qualitative accounts, though it was formally assessed with a validated instrument in only one study. Moreover, research engagement did not appear to protect students from burnout. Conclusion: Burnout and psychological distress are widespread among PhD students in medicine and health sciences, and impostor feelings emerged as a recurrent theme, although formally measured in only one study. These findings highlight the need for structural and institutional changes rather than relying solely on individual psychological support.

## 1. Introduction

In the past two decades, PhD education has undergone a profound transformation, driven by a range of interconnected factors. The growing number and diversity of doctoral candidates, the increasing value of knowledge and innovation in modern societies, and the expansion of PhD career pathways beyond academia have fundamentally transformed how doctoral education is conceptualized and implemented globally (Cardoso et al., 2022). Dropout rates of 30–50% in some countries and among different disciplines, prolonged and unpredictable completion times, and frequent reports of emotional distress raise serious questions about existing models of doctoral education (Satinsky et al., 2021; Vilser et al., 2024). These trends have been accompanied by increasing recognition that the psychological wellbeing of doctoral candidates requires systematic attention (Mahsood et al., 2025b; Martínez-García et al., 2024; Schmidt & Hansson, 2018). The intense competition and high expectations characteristic of doctoral training have contributed to a widely acknowledged mental health crisis in this population, with documented consequences ranging from reduced academic performance to elevated suicide risk (Bai et al., 2025). It is within this context that doctoral wellbeing has become a critical focus of research and institutional concern.

Numerous studies have shown that PhD students experience higher rates of psychological distress than the general population and their peers in other educational settings (Hazell et al., 2020; Satinsky et al., 2021). In a systematic review and meta-analysis which included 23,469 PhD students from multiple countries, Satinsky et al. found that approximately 24% experienced clinically significant symptoms of depression, while 17% suffered from anxiety. The authors concluded that systematic monitoring and promotion of mental health in this population is urgently needed (Satinsky et al., 2021). In a systematic review by Hazell et al. (2020), it was further confirmed that PhD students experience significantly higher levels of stress, identifying isolation and female gender as the most robust risk factors, while social support, positive supervisory relationships, self-care, and perceiving the PhD as a learning process emerged as the most well-established protective factors (Hazell et al., 2020). Similarly, Levecque et al. found that 32% of PhD students were at risk of developing a common psychiatric disorder, with work organization factors including supervisory style, job demands, and work–family interference identified as significant predictors of poor mental health (Levecque et al., 2017). Additionally, a global survey published by *Nature*, which included more than 6300 doctoral students from various regions, reported that over one-third of respondents (36%) sought help for anxiety or depression related to their PhD studies (Woolston, 2019), highlighting the prevalence of psychological difficulties among doctoral candidates worldwide. In a longitudinal study of 20,085 Swedish PhD students, Bergvall et al. analyzed administrative records of psychiatric medication use and found that by the fifth year of doctoral studies, psychiatric medication use had increased by 40% compared to pre-PhD levels, suggesting that doctoral training may contribute to the deterioration of students’ mental health (Bergvall et al., 2025).

Psychological distress can be understood as an emotional state marked by a perceived inability to cope with a stressor, accompanied by a shift in mood and a degree of harm that may be either short-lived or lasting (Ridner, 2004). Wellbeing, on the other hand, can be described as a state in which a person is able to draw on their abilities and stay in balance while dealing with everyday life demands (Galderisi et al., 2015). A high proportion of medical trainees experience significant stress, with key contributing factors including uncertainty about future careers in an increasingly competitive postgraduate market, difficult relationships with supervisors, and the feeling that their personal needs are neglected within the training system (Dyrbye & Shanafelt, 2016; Juliebø-Jones & Beisland, 2025). These structural characteristics can contribute to well-known risk factors for burnout, which have been extensively documented among medical students and residents (Cotobal Rodeles et al., 2025; Dyrbye & Shanafelt, 2016). Burnout is formally recognized under the code QD85 in the 11th revision of International Classification of Diseases (World Health Organization, n.d.), established by the World Health Organization between 2019 and 2021, as a syndrome resulting from chronic, unsuccessfully managed workplace stress, characterized by three dimensions: feelings of energy depletion or exhaustion, increased mental distance from one’s job or feelings of cynicism toward it, and a diminished sense of professional efficacy or accomplishment (World Health Organization, n.d.). The first standardized instrument for measuring burnout, the Maslach Burnout Inventory (MBI), was developed and validated by Maslach and Jackson (1981), and it became the dominant tool in this field of research. Given that students can also experience burnout, a student-specific version of the MBI was developed—the Maslach Burnout Inventory–Student Survey (MBI-SS)—reconceptualizing the same three dimensions in terms of study-related demands rather than occupational ones. This adaptation has been used to examine burnout in relation to student engagement and academic outcomes (Taris & Schaufeli, 2026). Van Vendeloo et al. found that residents experiencing burnout consistently perceived their learning environment more negatively than those without burnout, highlighting the quality of the learning environment as a key determinant of burnout (Van Vendeloo et al., 2018). Similarly, such structural factors may also play an important role within the context of PhD studies in medicine and health sciences. While much of the evidence above concerns either PhD students broadly or medical trainees without doctoral training, direct evidence specifically involving PhD students in medicine and health sciences is also emerging and converges on similarly high rates of psychological difficulty. Several studies have documented elevated burnout specifically within this population. Kusurkar et al. found that 53% of PhD students in medicine reported moderate or high levels of burnout, with doctoral students in clinical departments reporting significantly greater autonomy frustration and work-role conflict than those in non-clinical settings, though burnout itself did not differ significantly between the two groups (Kusurkar et al., 2021). Nagy et al. similarly reported that approximately half of biomedical doctoral students met criteria for at least one current psychiatric disorder (Nagy et al., 2019), and Tikkanen et al. identified distinct wellbeing profiles among PhD candidates in medicine and allied health fields, with high engagement consistently accompanied by at least moderate, rather than low, levels of burnout (Tikkanen et al., 2021). Qualitative findings worldwide further emphasize that supervisory and structural challenges are core issues within the doctoral experience in medicine and health sciences (Ahalli et al., 2022; Bazrafkan et al., 2016; den Bakker et al., 2024; Ghaseminangi et al., 2025; Mahsood et al., 2025a). These findings suggest that, although this population of PhD students share some vulnerabilities with the broader doctoral population, their dual research and clinical roles may introduce additional, context-specific pressures not fully captured by studies of either general PhD cohorts or non-doctoral medical trainees.

Beyond burnout, two additional constructs are particularly relevant to the PhD experience in this population. Impostor syndrome (IS), characterized by persistent self-doubt and fear of being exposed as a fraud despite evidence of competence, is highly prevalent among postgraduate medical professionals (Chua et al., 2025; Clance & Imes, 1978). Unlike burnout, IS does not hold formal classification status in diagnostic systems but remains a psychological construct most commonly assessed through validated self-report measures such as the Clance Impostor Phenomenon Scale (CIPS) (Clance, 1985). In a recent scoping review, Chua et al. found IS rates ranging from 23.5% to 75.9% among residents and clinicians and documented its association with anxiety, depression, burnout, suicidal ideation, and poorer overall wellbeing (Chua et al., 2025). In another scoping review, the authors identified several factors contributing to IS among all PhD students, including insufficient academic preparation, challenges related to role identity, a pervasive culture of genius in academic environments, and the influence of family background (Wang & Li, 2023). Alongside burnout and impostor syndrome, research engagement is another construct that has received increasing attention in higher education research. In this review, the focus is specifically on research engagement, given that research constitutes the core scholarly activity of doctoral training, rather than the broader construct of student engagement, which encompasses a wider range of academic activities beyond research itself. Research engagement reflects the extent to which PhD students actively and persistently invest themselves in the research process, shaping both their scholarly output and broader professional growth. Chen and Zhao demonstrated that academic passion significantly predicts the research engagement of PhD students, mediated by ambidextrous learning and the academic climate (Chen & Zhao, 2024). Stoeber et al. identified two types of passion for studying—harmonious and obsessive—and found that harmonious passion acts as a protective factor associated with higher academic engagement and lower burnout, while obsessive passion plays a more complex and context-dependent role (Stoeber et al., 2011). While burnout, impostor syndrome, and psychological distress (depression and anxiety) represent forms of negative psychological functioning, research engagement constitutes a conceptually distinct, motivational construct. Given its close relationship with these negative states among doctoral students, research engagement is included to capture the positive dimension of their psychological experience.

Despite the theoretical and practical relevance of these constructs, systematic research into the psychological outcomes of PhD students in medicine and health sciences remains limited. Existing literature reviews have focused on PhD candidates in general, without distinguishing the specific demands of medical doctoral studies (Satinsky et al., 2021); examined burnout among medical students and residents (Cotobal Rodeles et al., 2025; Dyrbye & Shanafelt, 2016); or investigated individual constructs in isolation, without exploring their interrelationships (Chua et al., 2025; Wang & Li, 2023). Consequently, it remains unclear to what extent burnout, IS, and research engagement are related within this specific population, and which key risk and protective factors are relevant to the context of medical doctoral education. This is particularly concerning given the unique pressures of PhD programs in this field, as psychological distress may have long-term consequences for research quality, workforce sustainability, and patient care. Considering these challenges, this systematic review aims to synthesize the available empirical evidence on burnout, IS, and research engagement among PhD students in medicine and health sciences. Specifically, we examine current knowledge, identify key risk and protective factors, and highlight gaps for future research, providing a foundation for the development of evidence-based interventions and institutional policies to support the psychological wellbeing of PhD candidates in medicine and health sciences.

## 2. Materials and Methods

### 2.1. Search Strategy

A systematic literature search was conducted in March 2026 across three electronic databases: Web of Science (WoS), Scopus, and Ovid MEDLINE. The search was limited to publications from 2015 onwards, in the English language, to capture evidence reflecting contemporary doctoral education structures and the substantial growth in scholarly and institutional attention to doctoral mental health over the past decade. This is corroborated by the distribution of the included literature itself: despite the ten-year search window, the majority of included studies (19 of 27) were published from 2021 onwards, indicating that relevant empirical research on this topic remained limited prior to this period.

For WoS and Scopus, an advanced search was applied combining three clusters with the Boolean operator AND, targeting titles, abstracts, and keywords (TS = for Web of Science; TITLE-ABS-KEY for Scopus): (1) population terms (PhD stud* OR doctoral stud* OR “MD-PhD” OR postgraduate stud*), (2) topic terms (passion OR motivation* OR perception* OR burnout OR impost* OR “academic performance”), and (3) field terms (biomed* OR medical OR “health science*” OR medicine).

For Ovid MEDLINE, the search was conducted in clusters using keyword fields (.ti,ab,kw.), combining population, engagement/motivation, field, and outcome terms with the Boolean operator AND: (“PhD stud**” OR “doctoral stud**” OR “MD-PhD” OR “master stud**” OR “postgraduate**” OR “PhD candidate**”) AND (passion OR motivation* OR perception* OR engagement OR commitment OR socioeconomic OR sociodemographic OR pressure) AND (medicine OR medical OR biomed* OR healthcare OR “health science**”) AND (burnout OR impost* OR “academic performance” OR “mental health” OR stress* OR anxiety OR depression OR wellbeing OR “well-being” OR productivity). The search strategy was expanded for Ovid MEDLINE by incorporating additional keywords and a dedicated outcome cluster to maximize sensitivity, as the initial criteria used for Web of Science and Scopus yielded an insufficient number of results for this specific database. Studies addressing research engagement were nonetheless identified through the WoS and Scopus searches via related terms such as ‘passion’ and ‘motivation’, which frequently co-occur with engagement-related outcomes in the titles, abstracts, and keywords of relevant publications, as well as through snowball sampling of reference lists. Free-text keyword searching was used across all three databases, rather than controlled vocabulary (e.g., MeSH terms), given the heterogeneity of terminology used across disciplines to describe the constructs of interest (e.g., burnout, impostor phenomenon, and research engagement). Broader population terms such as ‘master stud**’ and ‘postgraduate**’ were included in the Ovid MEDLINE strategy to further maximize sensitivity given the limited retrieval from this database; eligibility was subsequently determined independently of the search strategy through the title/abstract and full-text screening process, ensuring that only studies meeting the PhD/doctoral-specific inclusion criteria were retained regardless of which broader terms initially identified them.

### 2.2. Inclusion and Exclusion Criteria

Studies were included if they focused on PhD students or doctoral candidates in medicine and health sciences—while examining outcomes related to burnout, impostor syndrome, research engagement or passion, motivation for research, psychological wellbeing, or mental health. Furthermore, inclusion criteria encompassed both quantitative and qualitative research designs published in English from 2015 onwards with available full-text access. Given the inclusive eligibility approach adopted due to the limited literature specifically on PhD students in medicine and health sciences, some included studies involved broader postgraduate or professional populations where doctoral status could not always be precisely quantified.

Studies were excluded if they focused exclusively on populations other than PhD candidates, such as undergraduate students, or if they were not relevant to the topic of interest. Additionally, exclusion criteria applied to non-empirical studies including editorials, letters, commentaries, case reports, reviews, and conference abstracts lacking original data. Finally, papers were omitted if they were published before 2015, were unavailable in English, or if the full text could not be successfully retrieved.

### 2.3. Study Selection and Data Extraction

The search yielded 362 records from WoS, 228 from Ovid MEDLINE, and 125 from Scopus, totaling 715 records. After deduplication using Zotero (version 9.0.6) reference management software, 546 unique records were retained for screening. Title/abstract and full-text screening were both performed independently by two reviewers at each stage, using the predefined inclusion and exclusion criteria; disagreements were resolved through discussion until consensus was reached. Data extraction was likewise performed independently by two reviewers using a standardized Excel-based extraction form covering study characteristics, population, methodology, instruments, and key findings (summarized in Table 1, Table 2, Table 3, Table 4 and Table 5), with discrepancies similarly resolved through discussion. Where multiple included studies were based on overlapping or partially shared samples (Hish et al., 2019; Kusurkar et al., 2021, 2022; Nagy et al., 2019), each was retained as they addressed distinct research questions and reported non-redundant findings relevant to this review; this overlap is acknowledged when interpreting the cumulative sample size. Then, 312 records were excluded based on predefined exclusion criteria, and 234 records were sought for retrieval. Of these, 21 could not be retrieved, leaving 213 reports assessed for eligibility. After full-text review, an additional 190 records were excluded. To identify additional relevant studies, the reference lists of the included articles were manually screened (snowball sampling), resulting in four further eligible studies. The final result was a sample of 27 studies included in our synthesis. Due to the substantial heterogeneity in study designs, measurement instruments, and populations, a meta-analysis was not feasible, so findings were synthesized narratively.

**Figure 1:**
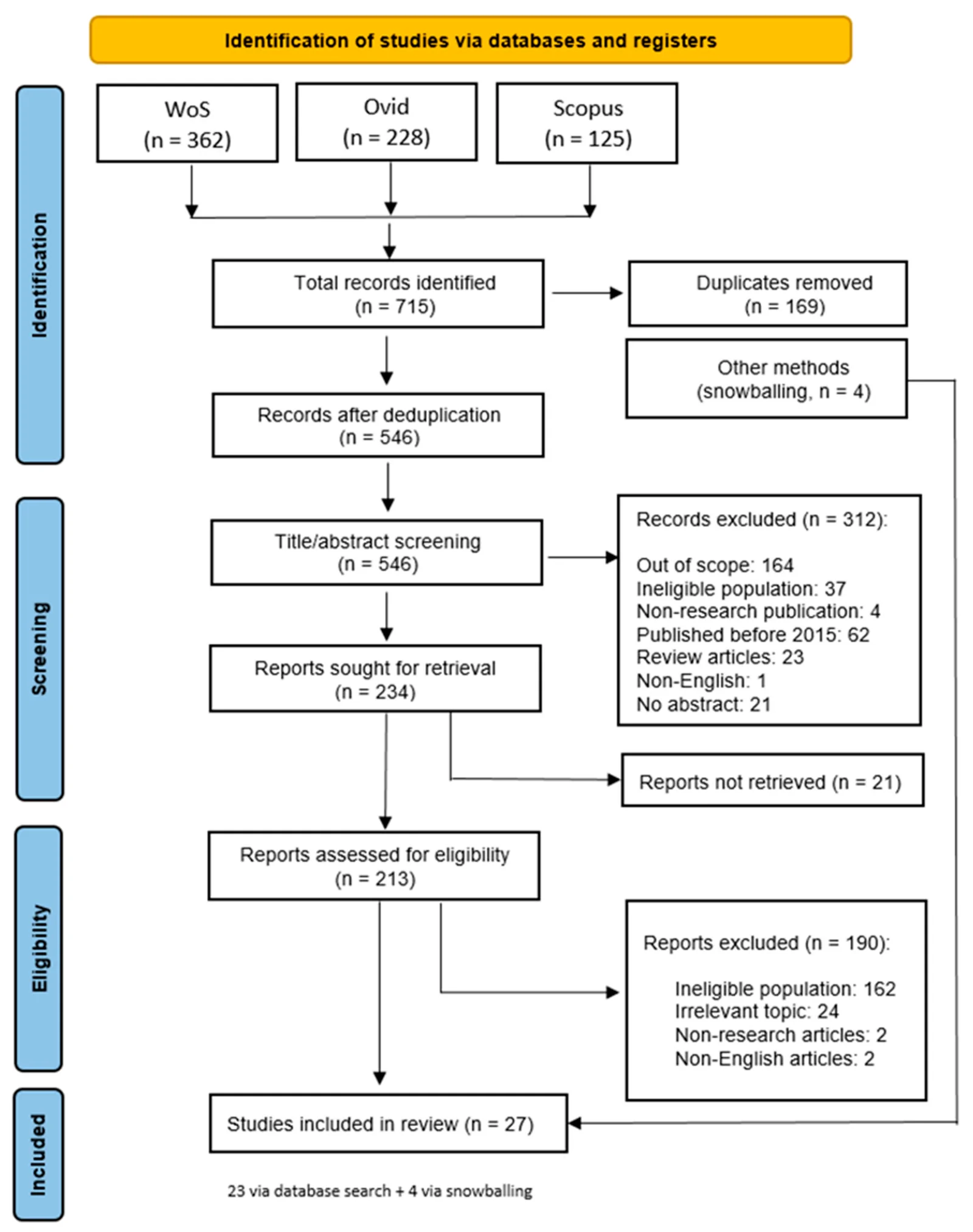
Flowchart of the systematic literature search and selection process according to PRISMA guidelines (Page et al., 2021).

This review was guided by the PEO framework: the population (P) comprised PhD students in medicine and health sciences; exposure (E) was defined as the demands and experiences of doctoral training; and the outcomes of interest (O) were burnout, impostor syndrome, research engagement, and related indicators of psychological distress. Depression, anxiety, and other distress-related outcomes were not treated as a separate, independently prespecified category but were synthesized alongside burnout given their substantial conceptual overlap and frequent co-assessment using the same or complementary instruments within the included studies. All decisions made during the screening process were documented, and reasons for exclusion were recorded for reporting in accordance with the PRISMA 2020 guidelines (Page et al., 2021). The rigorous selection process is visually summarized in the PRISMA flow diagram (Figure 1). All subsequent stages of the selection process, including the screening of titles, abstracts, and full texts, were performed manually by the authors without the aid of automation tools. Additionally, the review protocol was not prospectively registered.

### 2.4. Quality Appraisal

The methodological quality of the included studies was independently assessed by two reviewers using the Mixed Methods Appraisal Tool (MMAT, version 2018) (Hong et al., 2018), which enables appraisal of qualitative, quantitative non-randomized, quantitative descriptive, and mixed-methods studies within a single framework. Each study was first evaluated against two screening questions and then rated against five criteria specific to its study design category (Yes/No/Cannot tell). Discrepancies were resolved through discussion. For the two mixed-methods studies, the qualitative and quantitative components were each additionally appraised against the criteria of their respective methodological tradition, alongside the five mixed-methods-specific criteria, consistent with MMAT guidance (criterion 5.5). In line with MMAT recommendations, individual criterion ratings were not aggregated into a single overall score but instead reported descriptively by component (Table 6). Quality appraisal findings were not used to exclude studies but were considered when interpreting the strength of the synthesized evidence.

## 3. Results

### 3.1. Study Characteristics

A total of 27 studies met the inclusion criteria and were included in the synthesis of this systematic review. These studies were published over a ten-year period, reflecting increased research interest in recent years, as the majority (n = 19) were published from 2021 onwards. The total sample across all included studies approaches 7000 participants, with overlapping subsamples and studies reporting variable sample sizes across iterations taken into account (Chakraverty et al., 2022; Jachim et al., 2023; Kusurkar et al., 2021, 2022). The individual sample sizes are highly heterogeneous, ranging from small qualitative cohorts (Bazrafkan et al., 2016; Chakraverty et al., 2022; den Bakker et al., 2024; Howells et al., 2017; Mahsood et al., 2025a) to large multi-institutional cross-sectional surveys, with a maximum of 1645 participants (Tan et al., 2023).

Studies were conducted across five continents, suggesting that mental health challenges in doctoral education are widespread globally. Europe is the most represented continent, with nine studies conducted in countries including France (Ahalli et al., 2022), the Netherlands (den Bakker et al., 2024; Kusurkar et al., 2021, 2022), Belgium (Glorieux et al., 2025), Finland (Lonka et al., 2019), Sweden (Tikkanen et al., 2021), Portugal (Alves et al., 2025), Spain and Estonia (Prieto et al., 2022). Asia follows with seven studies from China (Gao et al., 2025; Ma et al., 2024; Tan et al., 2023), Iran (Bazrafkan et al., 2016; Ghaseminangi et al., 2025), Malaysia (Saleem et al., 2022), and Pakistan (Mahsood et al., 2025a). North America is represented by eight studies, all conducted in the USA (Chakraverty et al., 2022; Gin et al., 2021; Hish et al., 2019; Jachim et al., 2023; Nagy et al., 2019; Remich et al., 2016; Schad et al., 2022; White et al., 2018). The remaining studies were conducted across three continents: Africa (Matheka et al., 2020), South America (Souza et al., 2023), and Australia (Howells et al., 2017). Across the entire sample, the USA and China were the most frequently represented individual countries.

### 3.2. Methodological and Geographical Characteristics

Methodologically, the literature is dominated by cross-sectional survey designs, which account for 14 of the included studies (Ahalli et al., 2022; Gao et al., 2025; Hish et al., 2019; Jachim et al., 2023; Kusurkar et al., 2021; Lonka et al., 2019; Ma et al., 2024; Matheka et al., 2020; Nagy et al., 2019; Saleem et al., 2022; Schad et al., 2022; Souza et al., 2023; Tan et al., 2023; Tikkanen et al., 2021). Nine studies employed qualitative approaches, including descriptive, constructivist, and phenomenological designs (Alves et al., 2025; Bazrafkan et al., 2016; den Bakker et al., 2024; Ghaseminangi et al., 2025; Gin et al., 2021; Howells et al., 2017; Kusurkar et al., 2022; Mahsood et al., 2025a; Remich et al., 2016). Of the remaining studies, four were longitudinal (Glorieux et al., 2025; Jachim et al., 2023; Remich et al., 2016; White et al., 2018), three were intervention studies (Howells et al., 2017; Prieto et al., 2022; White et al., 2018), and two were mixed-methods studies (Chakraverty et al., 2022; Prieto et al., 2022). Five studies fall into more than one methodological category (Table 5): Jachim et al. was both cross-sectional and longitudinal (Jachim et al., 2023); Howells et al. was both qualitative and an intervention study (Howells et al., 2017); Prieto et al. was both mixed-methods and an intervention study (Prieto et al., 2022); Remich et al. was both longitudinal and qualitative (Remich et al., 2016); and White et al. was both longitudinal and an intervention study (White et al., 2018). Most samples focused exclusively on PhD candidates or doctoral scholars (n = 18) (Ahalli et al., 2022; Bazrafkan et al., 2016; den Bakker et al., 2024; Ghaseminangi et al., 2025; Gin et al., 2021; Glorieux et al., 2025; Hish et al., 2019; Howells et al., 2017; Kusurkar et al., 2021, 2022; Lonka et al., 2019; Mahsood et al., 2025a; Matheka et al., 2020; Nagy et al., 2019; Prieto et al., 2022; Remich et al., 2016; Saleem et al., 2022; Tikkanen et al., 2021), while four studies included both master’s and doctoral students (Gao et al., 2025; Ma et al., 2024; Souza et al., 2023; Tan et al., 2023), two recruited MD-PhD students or medical residents (Chakraverty et al., 2022; Schad et al., 2022), and three included mixed samples also comprising supervisors, master’s students, or postgraduate public health students (Alves et al., 2025; Jachim et al., 2023; White et al., 2018). Female participants constitute the dominant demographic in nearly all samples, with their proportion ranging from 40% (Matheka et al., 2020) to 100% in the female-only cohort (Remich et al., 2016), while most mixed samples are between 52% and 81% female representation, with some studies reporting notably lower proportions: 42% (Ahalli et al., 2022) and 44% (Saleem et al., 2022). Burnout was most frequently assessed with the Maslach Burnout Inventory (MBI) in its student and medical personnel formats (MBI-SS, MBI-HSS-MP; n = 2) (Gao et al., 2025; Kusurkar et al., 2021), the School Burnout Inventory (SBI; n = 2) (Hish et al., 2019; Nagy et al., 2019), and the Cross-Country Doctoral Experience Survey exhaustion and cynicism subscales (C-DES; n = 1) (Tikkanen et al., 2021). Research and academic engagement were tracked primarily with the Utrecht Work Engagement Scale or its student variants (UWES/UWES-S-9; n = 2) (Kusurkar et al., 2021; Saleem et al., 2022) and the C-DES engagement subscale (n = 1) (Tikkanen et al., 2021). The Clance Impostor Phenomenon Scale (CIPS) was the only quantitative measure used to assess the impostor phenomenon (Chakraverty et al., 2022). General mental health and distress outcomes were mainly assessed with the Patient Health Questionnaire (PHQ-9; n = 4) (Ahalli et al., 2022; Hish et al., 2019; Nagy et al., 2019; Schad et al., 2022), the Generalized Anxiety Disorder scale (GAD-7; n = 3) (Ahalli et al., 2022; Jachim et al., 2023; Schad et al., 2022), the Center for Epidemiologic Studies Depression Scale (CESD-R; n = 1) (Jachim et al., 2023), and the Depression Anxiety Stress Scale (DASS-21; n = 2) (Ma et al., 2024; Saleem et al., 2022). Notably, one study employed structured clinical interviews—the Structured Clinical Interview for DSM-5 Research Version (SCID-5-RV) and the Structured Clinical Interview for DSM-5 Personality Disorders (SCID-5-PD)—rather than self-report measures alone (Nagy et al., 2019), and one used a burnout scale developed specifically for the doctoral context (Prieto et al., 2022). The detailed characteristics of each individual study are summarized in Table 1. The geographical and demographic mapping of the 27 included studies reveals a clear pattern in how the mental health and wellbeing of PhD candidates is currently investigated. A significant proportion of the included studies originate from Western academic systems (n = 19; 70%), with the United States and the Netherlands being the most represented countries (Ahalli et al., 2022; Alves et al., 2025; Chakraverty et al., 2022; den Bakker et al., 2024; Gin et al., 2021; Glorieux et al., 2025; Hish et al., 2019; Howells et al., 2017; Jachim et al., 2023; Kusurkar et al., 2021, 2022; Lonka et al., 2019; Nagy et al., 2019; Prieto et al., 2022; Remich et al., 2016; Saleem et al., 2022; Schad et al., 2022; Tikkanen et al., 2021; White et al., 2018). In contrast, only eight studies were conducted outside this context, representing China, Iran, Brazil, Kenya, and Pakistan (Bazrafkan et al., 2016; Gao et al., 2025; Ghaseminangi et al., 2025; Ma et al., 2024; Mahsood et al., 2025a; Matheka et al., 2020; Souza et al., 2023; Tan et al., 2023). Notably, qualitative studies offered valuable insights into non-Western and transitional academic contexts, particularly Iran (Bazrafkan et al., 2016; Ghaseminangi et al., 2025) and Pakistan (Mahsood et al., 2025a), and highlighted structural and socioeconomic stressors often absent from the Western literature. A similar focus on structural factors was evident in Kenya, where Matheka et al. examined doctoral progress within the African academic context (Matheka et al., 2020). Finally, the methodological landscape was notably lacking in longitudinal and intervention-based research. The three intervention studies (Howells et al., 2017; Prieto et al., 2022; White et al., 2018) were all limited by small sample sizes and the absence of randomized control groups.

### 3.3. Burnout and Mental Health Outcomes

The evidence shows how burnout affects a substantial proportion of PhD candidates in medical and health sciences. Kusurkar et al. found that 53% of PhD students in medicine reported moderate or high levels of burnout (Kusurkar et al., 2021), and Nagy et al. reported that approximately half of biomedical doctoral students met criteria for at least one current psychiatric disorder (Nagy et al., 2019). Rates of depression and anxiety were also considerable, ranging from 21% to 28% for depression and from 9% to 38% for anxiety across studies (Jachim et al., 2023; Ma et al., 2024; Tan et al., 2023). These percentages should be interpreted with caution, as they were derived from different instruments with varying thresholds and psychometric properties, rather than directly comparable measures of a single construct. Notably, Schad et al. found that biomedical PhD students reported significantly higher rates of depression, anxiety, and suicidal ideation compared to medical students at the same institution (Schad et al., 2022). Burnout, depression, and anxiety among doctoral candidates in medical and health sciences are strongly shaped by the institutional and departmental context in which students work. Kusurkar et al. found that doctoral students working in clinical environments face significantly greater autonomy frustration and conflict between work responsibilities than those in non-clinical settings (Kusurkar et al., 2021), though burnout itself did not differ significantly between the two groups. Interestingly, Tikkanen et al. reported that working in a clinical unit or research group was associated with lower burnout and higher engagement (Tikkanen et al., 2021), possibly reflecting differences in institutional context and how clinical work is structured across settings. Notably, the same study identified professional isolation as an even stronger predictor of poor wellbeing, with doctoral candidates working alone being significantly more likely to fall into the worst wellbeing profiles compared to those working within a research group (OR = 4.22) (Tikkanen et al., 2021). Hish et al. further demonstrated that different types of stress predict different outcomes—academic stress drives burnout, while financial and family pressures are more closely associated with depression (Hish et al., 2019). A substantial treatment gap was also identified, with Jachim et al. reporting that between 40% and 47% of students with symptoms of depression or anxiety did not seek professional mental healthcare (Jachim et al., 2023).

### 3.4. Impostor Phenomenon and Impostor Feelings in PhD Students

Several included studies identified impostor feelings as a significant psychological burden among PhD candidates in biomedical and life sciences. Gin et al. reported that some doctoral students experiencing depression described feeling like impostors within a broader qualitative theme of low self-esteem and self-criticism, which affected 58% of their sample (Gin et al., 2021). The development of these feelings is closely related to the underlying mental models that students hold about their academic journey. Qualitative findings by den Bakker et al. suggest that impostor feelings are more likely to emerge when candidates feel they must constantly prove they deserve their place in the program, rather than viewing it as a process of learning and development (den Bakker et al., 2024). Furthermore, the intensity of this experience is not uniform across demographic groups, at least among MD-PhD candidates. Chakraverty et al. found a moderate-to-intense impostor phenomenon among all nine interviewed MD-PhD participants—a small, self-selected sample recruited specifically because they had already identified experiencing impostor phenomenon—with underrepresented minority students reporting particularly intense experiences, including a sense of ‘othering’ within academia (Chakraverty et al., 2022). Finally, impostor feelings appear to be most prominent in the early phases of doctoral training, suggesting that the transition into the program represents a particularly vulnerable period (den Bakker et al., 2024).

### 3.5. Research Engagement and Wellbeing

Evidence on research engagement revealed a more complex situation than might be expected, as high engagement does not necessarily protect against burnout or psychological distress. Tikkanen et al. found that medical doctoral programs are highly engaging, with moderate to high engagement recorded across all identified wellbeing profiles; however, high engagement was consistently paired only with low or moderate—never high—burnout, while profiles marked by high burnout involved moderate, not high, engagement (Tikkanen et al., 2021). Similarly, Kusurkar et al. found that autonomous motivation and satisfaction of basic psychological needs were key drivers of research engagement among PhD students in medicine (Kusurkar et al., 2021). A subsequent qualitative study by the same research group identified several sources of energy in doctoral work, with relatedness with colleagues being the most frequently cited energizer across the entire qualitative sample. Conversely, within the categories of time pressure and career uncertainty, the same study found no energizers—only stressors, most notably contract expiry (Kusurkar et al., 2022). Teaching activities also emerged as a potential source of psychological support. Gin et al. found that PhD students experiencing depression more often reported improvement in their mental state through teaching than through research, largely due to the structure and positive reinforcement that teaching provided (Gin et al., 2021). Saleem et al. further found that positive emotions significantly increased academic engagement among PhD students, while stress negatively moderated this relationship—suggesting that emotional wellbeing plays an important role in sustaining research engagement (Saleem et al., 2022).

### 3.6. Protective Factors and Structural Barriers

Several included studies suggest that removing structural barriers may be as important for doctoral wellbeing as promoting individual psychological resources. Grounded in Self-Determination Theory, Kusurkar et al. found that preventing the frustration of basic psychological needs—autonomy, competence, and relatedness—matters more for avoiding burnout than ensuring their satisfaction, suggesting that institutions should focus on reducing conditions that actively undermine these needs. The same study also identified sleep quality and work–life balance as significant protective factors against burnout (Kusurkar et al., 2021). The quality of the student–supervisor relationship consistently emerged as a key protective factor across the reviewed studies. Ahalli et al. found that a poor supervisory relationship is among the primary predictors of mental health difficulties (Ahalli et al., 2022), while den Bakker et al. highlighted supervision quality as a key moderator of PhD engagement (den Bakker et al., 2024). Chakraverty et al. proposed closed mentoring triads as a possible protective mechanism (Chakraverty et al., 2022), while Ghaseminangi et al. and Mahsood et al. emphasized peer support and supervisor availability as important buffers, with Ghaseminangi also highlighting the role of transparent institutional procedures (Ghaseminangi et al., 2025; Mahsood et al., 2025a).

### 3.7. Quality Appraisal

Overall, the methodological quality of the included studies was mixed (Table 6). Among the nine qualitative studies, quality was generally high, with six meeting all five applicable criteria and the remaining three meeting four of five, most commonly reflecting limited transparency in how findings were derived from raw data (e.g., absence of independent double-coding). Among the 16 quantitative descriptive and non-randomized studies, quality was more variable: three studies (Ahalli et al., 2022; Gao et al., 2025; Tan et al., 2023) met all five criteria, while the majority (n = 10) met three of five, most frequently due to unclear or non-representative sampling and an inability to rule out nonresponse bias given moderate response rates that varied considerably across studies (ranging from approximately 12 to 47%) and were often difficult to verify due to the anonymous nature of data collection. The single intervention study assessed within this category (White et al., 2018) met only two of five criteria, reflecting the absence of a control group and limited outcome measurement. The quantitative components of the two mixed-methods studies (Chakraverty et al., 2022; Prieto et al., 2022) showed the most pronounced limitations, meeting only two of five criteria each, primarily due to small, self-selected samples and, in the case of Chakraverty et al. (2022), the absence of formal integration between qualitative and quantitative components (0 of 5 mixed-methods-specific criteria met). Full appraisal ratings are provided in Table 6.

## 4. Discussion

This review included 27 studies examining burnout, impostor syndrome, research engagement, and mental health outcomes among PhD students in medicine and health sciences. Across the included studies, psychological difficulties among PhD candidates appear to be driven more by institutional and relational factors than by individual characteristics. Burnout was the most extensively studied construct, with elevated prevalence reported across diverse national and institutional contexts. Impostor syndrome was formally measured using a validated instrument in only one study, yet impostor feelings appeared repeatedly in qualitative accounts across several others. Research engagement presented a paradoxical pattern, as high engagement did not preclude at least moderate levels of burnout, suggesting that engagement alone does not necessarily protect against psychological distress. Across all examined constructs, the quality of the supervisory relationship and the broader learning environment emerged as the most consistent determinants of doctoral wellbeing, a pattern observed across different countries, study designs, and outcome measures.

The reviewed evidence confirms that burnout is widespread among PhD students in medicine and health sciences, and that it is more strongly associated with structural and institutional conditions than with individual vulnerability. Professional isolation emerged as a particularly strong risk factor. Tikkanen et al. found that working outside a research group substantially increased the likelihood of poor wellbeing profiles (Tikkanen et al., 2021), a pattern also noted in several qualitative studies (Bazrafkan et al., 2016; Ghaseminangi et al., 2025; Gin et al., 2021; Kusurkar et al., 2022; Mahsood et al., 2025a). The included studies also suggest that different types of stressors lead to different psychological outcomes. Academic stressors are more closely tied to burnout, while financial and family pressures are more strongly associated with depression (Hish et al., 2019). This suggests that institutions may need different types of support for different psychological outcomes, rather than a uniform approach. Depression and anxiety rates varied considerably across the reviewed studies, with depression ranging from approximately 10% to 65% and anxiety from approximately 9% to 67%, depending on the population, context, and time of data collection (Jachim et al., 2023; Nagy et al., 2019; Schad et al., 2022; Tan et al., 2023). This wide variation partly reflects differences in the measurement instruments used across studies rather than solely true differences in prevalence, limiting the direct comparability of these estimates. The upper bounds of these ranges were reported by Schad et al., whose data were collected during the COVID-19 pandemic, which may have contributed to the exceptionally high rates observed (Schad et al., 2022). A substantial proportion of students with symptoms of depression or anxiety did not seek professional help (Jachim et al., 2023), pointing to a treatment gap that calls for more proactive institutional responses.

This review identified a notable lack of formal research on impostor syndrome among PhD students in medicine and health sciences. Despite being explicitly named in a small number of qualitative studies (Chakraverty et al., 2022; den Bakker et al., 2024; Gin et al., 2021), impostor syndrome was formally measured using a validated instrument in only one included study (Chakraverty et al., 2022), which employed a mixed-methods design. Given this limited evidence base, findings regarding the prevalence, subgroup differences, or determinants of impostor phenomenon discussed below should be interpreted as exploratory and hypothesis-generating rather than established. Qualitative evidence suggests that whether doctoral candidates view the PhD as a test of their worth or as a learning process may influence whether impostor feelings develop (den Bakker et al., 2024). This points to a meaningful role for supervisors and institutions in shaping the psychological environment of doctoral training, not only through formal support structures but also through the expectations they set about what the PhD is and should be (den Bakker et al., 2024; Kusurkar et al., 2021, 2022). In the single study that included underrepresented minority participants, impostor experiences were described as particularly intense; however, given the small, self-selected qualitative sample, this pattern should be regarded as exploratory rather than an established determinant. Furthermore, the included studies suggest that PhD candidates in medicine and health sciences generally report high levels of research engagement, yet this does not appear to protect against burnout or psychological distress. Even high engagement was consistently accompanied by at least moderate burnout, suggesting that high research engagement alone is not sufficient to sustain wellbeing (Tikkanen et al., 2021). Self-Determination Theory, as applied by Kusurkar et al., offers a useful framework for understanding this pattern (Kusurkar et al., 2021). The reviewed evidence suggests that the type of motivation matters as much as its intensity. Students who are driven by genuine interest in their research and a sense of personal meaning tend to show better wellbeing outcomes than those who are driven primarily by external pressures or fear of failure (Kusurkar et al., 2021, 2022). This has practical implications for how PhD programs are structured, how supervisors communicate expectations, and how institutions present the purpose of doctoral training.

Teaching activities emerged as a potential source of psychological support, particularly for students experiencing depression. The structure and positive reinforcement of teaching appeared to help students manage depressive symptoms, suggesting that structured non-research activities may offer some psychological benefit within PhD programs (Gin et al., 2021). Moreover, the quality of the supervisory relationship emerges as the most consistently reported factor influencing PhD wellbeing (Ahalli et al., 2022; Bazrafkan et al., 2016; den Bakker et al., 2024; Glorieux et al., 2025; Hish et al., 2019; Kusurkar et al., 2022; Mahsood et al., 2025a). Poor supervision was associated with depression, anxiety, dropout, and burnout across multiple studies and was also qualitatively linked to impostor feelings in a subset of these studies (Ahalli et al., 2022; den Bakker et al., 2024; Glorieux et al., 2025; Hish et al., 2019; Kusurkar et al., 2021), while good supervision was among the most frequently cited sources of motivation and psychological support (Howells et al., 2017; Kusurkar et al., 2021). This consistency across diverse contexts—from the Netherlands and Sweden to Iran, Pakistan, and Kenya—suggests that supervisory quality should be treated as an institutional responsibility rather than a private matter between student and supervisor (Bazrafkan et al., 2016; Ghaseminangi et al., 2025; Kusurkar et al., 2021; Mahsood et al., 2025a; Matheka et al., 2020; Tikkanen et al., 2021). The evidence points toward several structural approaches, including clear institutional procedures for students experiencing difficulties (Ghaseminangi et al., 2025; Mahsood et al., 2025a) and involving closed mentoring triads so that students are not entirely dependent on a single person (Chakraverty et al., 2022). Nevertheless, encouraging good supervision without institutional structures to support it is unlikely to produce consistent change. Beyond the supervisory relationship, the broader learning environment also matters. Peer support, research group membership, and a sense of belonging to an academic community emerged as meaningful protective factors across several included studies (Ghaseminangi et al., 2025; Gin et al., 2021; Kusurkar et al., 2021; Mahsood et al., 2025a; Tikkanen et al., 2021).

Female PhD candidates reported higher levels of psychological distress in several included studies (Ghaseminangi et al., 2025; Jachim et al., 2023; Mahsood et al., 2025a; Schad et al., 2022), though this pattern was not consistent across all outcomes and contexts. This pattern appears to reflect not only the pressures of doctoral training itself but also the additional challenges, including the pressure to balance research productivity with family responsibilities (Ghaseminangi et al., 2025; Mahsood et al., 2025a), the experience of gender bias in academic environments (Remich et al., 2016; Schad et al., 2022), and managing gender identity in academic settings (Remich et al., 2016), which together contribute to an additional psychological burden among female PhD candidates. This was particularly evident in studies from non-Western settings, where cultural norms around gender roles compound the already significant demands of doctoral training (Ghaseminangi et al., 2025; Mahsood et al., 2025a).

The included studies also revealed that the sources of PhD distress vary considerably across different national and institutional contexts. Studies from Western academic systems tend to focus on psychological and motivational factors (den Bakker et al., 2024; Kusurkar et al., 2021; Tikkanen et al., 2021), while studies from Africa, the Middle East, and South Asia more consistently highlight structural and socioeconomic barriers—including funding insecurity, institutional instability, and policy gaps—as key drivers of doctoral distress (Bazrafkan et al., 2016; Ghaseminangi et al., 2025; Mahsood et al., 2025a; Matheka et al., 2020). Support programs developed in Western universities may therefore not translate directly to other contexts, particularly settings where doctoral students face primarily structural and financial barriers and where institutional and policy changes may be equally important as individual psychological support (Ghaseminangi et al., 2025; Mahsood et al., 2025a).

This review has several notable strengths and limitations. While previous systematic reviews have examined burnout or mental health among PhD students more broadly, this is the first to focus specifically on the intersection of burnout, impostor syndrome, and research engagement within the population of PhD students in medicine and health sciences. The inclusion of both quantitative and qualitative studies enabled a more complete picture of the evidence, capturing the prevalence of psychological difficulties and the contextual factors and personal experiences that contribute to them. Research was conducted across five continents and a wide range of academic systems, which strengthens the broader relevance of the findings. The screening and data extraction process were performed independently by two reviewers, with disagreements resolved through discussion, reducing the risk of bias in study selection and data interpretation. However, studies relying on self-reported measures may not fully capture the true prevalence of mental health problems among doctoral students, as administrative data from Swedish PhD cohorts suggest considerably lower rates than those reported in survey-based research (Keloharju et al., 2024). Considerable heterogeneity in study designs, measurement instruments, and populations made meta-analysis impossible and limits the direct comparability of findings. Some studies included mixed samples—combining students from different disciplines or from both master’s and doctoral programs—which may limit how specifically the findings apply to PhD students in medicine and health sciences. In addition, closer examination of a small number of included studies revealed that some samples labeled as ‘postgraduate’ in the original publications may in fact correspond predominantly to master’s-level, professional, or residency training programs rather than research doctorates, and in some cases only a minority of participants belonged to health-related disciplines; such heterogeneity in how ‘postgraduate’ or ‘doctoral’ status was defined and reported across studies further limits the precision, though not necessarily the direction, of the synthesized findings. The prevalence of cross-sectional designs means that causal relationships cannot be established, and how burnout, engagement, and impostor syndrome develop and change over the course of doctoral training remains poorly understood. Quality appraisal using the MMAT (Table 6) further indicated that the two studies employing quantitative measures of impostor syndrome (Chakraverty et al., 2022) and intervention outcomes (Prieto et al., 2022) showed the most pronounced methodological limitations among the included studies, primarily reflecting small, self-selected samples; this reinforces the exploratory interpretation of findings regarding impostor syndrome prevalence and intervention effectiveness discussed above. Finally, it is possible that studies with non-significant or negative findings were less likely to be published, which may have influenced the overall picture presented in this review.

The findings of this review point to several practical priorities for institutions involved in PhD education in medicine and health sciences. As discussed above, supervisory quality should be treated as an institutional responsibility, rather than left to individual relationships. Reducing professional isolation, as discussed above, also represents a well-supported priority. The limited quantitative research on impostor syndrome in this population is a clear gap that future studies should address, using validated tools such as the Clance Impostor Phenomenon Scale to examine its prevalence, predictors, and consequences in larger and more representative samples. Longitudinal study designs are also needed to understand how burnout, engagement, and impostor syndrome develop across different PhD phases and how early risk factors predict later outcomes. Intervention studies with adequate sample sizes and appropriate control conditions remain largely absent from the current literature and represent an important gap. Finally, the evidence from non-Western contexts calls for greater attention to structural and socioeconomic determinants of PhD wellbeing in both research and policy. Individually focused psychological interventions may be insufficient in settings where institutional instability, funding insecurity, and policy gaps are the primary drivers of PhD distress. Improving the wellbeing of PhD students in medicine and health sciences will require both better support within universities and broader changes to the conditions of doctoral training.

## 5. Conclusions

This review demonstrates that burnout and psychological distress are widespread among doctoral students in medicine and health sciences, and that impostor feelings emerged as a recurrent theme in qualitative accounts, though impostor syndrome was formally assessed with a validated instrument in only one included study. These difficulties appear to be more strongly associated with institutional and relational factors than with individual characteristics alone. Notably, research engagement did not appear to protect against these difficulties, as even highly engaged doctoral candidates were not free of at least moderate burnout. The quality of the supervisory relationship, the degree of professional isolation, and the broader learning environment consistently emerged as the factors most strongly associated with doctoral wellbeing across diverse national and cultural contexts. These findings suggest that improving doctoral wellbeing requires structural and institutional change, not only individual-level support. Future research should prioritize longitudinal designs, validated measurements of impostor syndrome, and intervention studies with stronger methodological designs.

## Figures and Tables

**Table 1:** Characteristics of included studies (n = 27).

Authors (Year)	Country	N	Female (%)	Study Design	Population	Key Instruments
Ahalli et al. (2022)	France	161	42	Cross-sectional (occupational health monitoring)	1st-year PhD students, health and science disciplines; doctoral contract	JCQ; AWAI-S; GAD-7; PHQ-9
Alves et al. (2025)	Portugal	105	59	Qualitative	PhD candidates, holders, supervisors, SMC members; social and health sciences	Focus groups
Bazrafkan et al. (2016)	Iran	16	NR	Qualitative (descriptive)	Medical sciences PhD students during thesis writing; 4 universities	Semi-structured interviews; field observations
den Bakker et al. (2024)	Netherlands	10	NR	Qualitative (constructivist)	Medical PhD candidates in final phase of PhD	Timeline-assisted semi-structured interviews
Chakraverty et al. (2022)	USA	13	NR	Mixed-methods	MD-PhD students and residents; 7 MD-PhD programs	CIPS; online survey + semi-structured interviews
Gao et al. (2025)	China (Southern)	315	68	Cross-sectional	Clinical postgraduate dental students (3-year professional degree); 8 South Chinese institutions; multiple specialties	Online survey; MBI-HSS-MP; ERQ; DPSI
Ghaseminangi et al. (2025)	Iran	31	61.3	Qualitative (descriptive)	PhD candidates in medical sciences; ≥2 years enrolled	Semi-structured interviews
Gin et al. (2021)	USA	50	58	Qualitative	Life sciences PhD students with self-identified depression; 28 institutions	Semi-structured interviews
Glorieux et al. (2025)	Belgium	589 (101 Life Sciences & Medicine)	52	Longitudinal (4 survey waves)	First-year PhD students, Vrije Universiteit Brussel	TPS; SSS; PRI
Hish et al. (2019) *	USA	69	61	Cross-sectional (pilot study)	Biomedical PhD students, Duke University	GSI-R; SBI; PHQ-9; PMS; MSPSS; AWAI
Howells et al. (2017)	Australia	8	NR	Qualitative case study (6-week)	PhD students in chemistry and pharmacy, University of Tasmania	Semi-structured interviews
Jachim et al. (2023)	USA	306–364	57–64	Cross-sectional; three time points (2019, 2020, 2021)	Biomedical PhD, master’s and MD/PhD students (in PhD phase), Mayo Clinic Graduate School	CESD-R; GAD-7; Satisfaction with Life Scale; ISEL-12
Kusurkar et al. (2021) †	Netherlands	464	80	Cross-sectional	PhD students in medicine, Amsterdam UMC	MBI-SS; UWES-S-9; SRQ-A; BPNS; WLB Scale
Kusurkar et al. (2022) †	Netherlands	386	81	Qualitative	PhD students in medicine, Amsterdam UMC	Open-ended survey questions (top 3 stressors and energizers)
Lonka et al. (2019)	Finland	664	75	Cross-sectional (person-centred)	PhD students, University of Helsinki (arts, medicine, educational sciences)	WPQ; MED NORD adapted; PLE
Nagy et al. (2019) *	USA	69	61	Cross-sectional (pilot study)	Biomedical PhD students, Duke University; mean age 26.5 yrs; years 1–6 of training	SBI; PHQ-9; SCID-5-RV; SCID-5-PD; WSAS; EOI; GPCS; RTES-R
Ma et al. (2024)	China	530	66	Cross-sectional	Medical postgraduate students, Shanxi Medical University	AGOS (MAP, PAP, MAV, PAV); SES; CPSS; DASS-21
Matheka et al. (2020)	Kenya	628 (96 medical sciences)	40	Cross-sectional	PhD students, Kenyan universities; enrolled 2010–2018; medical sciences subgroup	MSLQ adapted (intrinsic + extrinsic motivation); GSES
Mahsood et al. (2025a)	Pakistan	11	82	Qualitative (phenomenological)	Medical & allied health sciences PhD scholars; 9 Pakistani universities	Semi-structured in-depth interviews
Souza et al. (2023)	Brazil	117	80	Cross-sectional	Postgraduate healthcare students (master’s + doctoral), Mato Grosso state, Brazil	DSM-5 SALS-A; sociodemographic questionnaire
Prieto et al. (2022)	Spain & Estonia	82	50–79	Mixed-methods (design-based research, 4 iterations, 5 workshops)	PhD students from multiple disciplines	CPC-12 (PsyCap); doctoral burnout scale
Remich et al. (2016)	USA	22	100	Longitudinal qualitative (descriptive)	Biomedical PhD students; 16 institutions	Annual interviews
Saleem et al. (2022)	Malaysia	373	44	Cross-sectional	PhD students, 23 private Malaysian universities; multiple disciplines; health sciences 8% of sample	SPANE; PCQ-12; UWES (adapted); DASS-21 (stress subscale)
Schad et al. (2022)	USA	957 (309 PhD, 622 MD)	67	Cross-sectional; two time points (2019, 2020)	Biomedical PhD students and medical students, UNC Chapel Hill	PHQ-9; GAD-7; AUDIT; DAST-10; suicidal ideation
Tan et al. (2023)	China	1,645	61	Cross-sectional	Medical postgraduate students (master’s + doctoral), Southern Medical University, Guangdong	SDS; SAS; QPSP; SCSQ; SSRS
Tikkanen et al. (2021)	Sweden	692	61	Cross-sectional (person-centered)	PhD candidates in medicine and allied health fields; Karolinska Institutet	C-DES (research engagement, exhaustion, cynicism subscales)
White et al. (2018)	USA	91	74	Longitudinal pre–post intervention study	Postgraduate public health students	BRFSS; HRQOL; SF-36

AGOS = Achievement Goal Orientation Scale (subscales: MAP = mastery-approach goal, MAV = mastery-avoidance goal, PAP = performance-approach goal, PAV = performance-avoidance goal); AUDIT = Alcohol Use Disorder Identification Test; AWAI = Advisory Working Alliance Inventory; AWAI-S = Advisory Working Alliance Inventory—Student version; BPNS = Basic Psychological Needs Scale; BRFSS = Behavioral Risk Factor Surveillance System; C-DES = Cross-Country Doctoral Experience Survey; CESD-R = Center for Epidemiologic Studies Depression Scale—Revised; CIPS = Clance Impostor Phenomenon Scale; CPC-12 = Compound PsyCap Scale-12; CPSS = Chinese Perceived Stress Scale; DASS-21 = Depression Anxiety Stress Scale-21; DAST-10 = Drug Abuse Screening Test-10; DBR = Design-Based Research; DPSI = Dental Postgraduate Stress Inventory; DSM-5 SALS-A = DSM-5 Self-Applicable Level 1 Symptoms Scale—Adult; EOI = Employment Opportunity Index; ERQ = Emotion Regulation Questionnaire; GAD-7 = Generalized Anxiety Disorder-7; GPCS = Graduate Program Climate Scale; GSES = General Self-Efficacy Scale; GSI-R = Graduate Stress Inventory-Revised; HRQOL = Health-Related Quality of Life Assessment; ISEL-12 = Interpersonal Support Evaluation List (12-item); JCQ = Job Content Questionnaire; MBI-HSS-MP = Maslach Burnout Inventory—Human Services Survey for Medical Personnel; MBI-SS = Maslach Burnout Inventory—Student Survey; MED NORD = Nordic Well-Being Questionnaire adapted for doctoral context; MSLQ = Motivated Strategies for Learning Questionnaire; MSPSS = Multidimensional Scale of Perceived Social Support; NR = not reported; PCQ-12 = Psychological Capital Questionnaire-12; PHQ-9 = Patient Health Questionnaire-9; PLE = Perceptions of the Learning Environment; PMS = Pearlin Mastery Scale; PRI = Passion for Research Index (single item); QPSP = Questionnaire on Psychological Stressors of Postgraduates; RTES-R = Research Training Environment Scale—Revised; SAS = Self-rating Anxiety Scale; SBI = School Burnout Inventory; SCID-5-PD = Structured Clinical Interview for DSM-5 Personality Disorders; SCID-5-RV = Structured Clinical Interview for DSM-5, Research Version; SCSQ = Simplified Coping Style Questionnaire; SDS = Self-rating Depression Scale;; SES = Rosenberg Self-Esteem Scale; SF-36 = Short Form-36; SMC = Members of scientific and monitoring committees; SPANE = Scale of Positive and Negative Experiences; SRQ-A = Academic Self-Regulation Questionnaire; SSS = Supervisor Support Scale; SSRS = Social Support Rate Scale; TPS = Time Pressure Scale; UWES = Utrecht Work Engagement Scale; UWES-S-9 = Utrecht Work Engagement Scale—Student version (9-item); WLB Scale = Work-Life Balance Scale; WPQ = Writing Process Questionnaire; WSAS = Work and Social Adjustment Scale. Note. Several studies included mixed or multidisciplinary samples (Ahalli et al., 2022; Alves et al., 2025; Glorieux et al., 2025; Lonka et al., 2019; Matheka et al., 2020; Prieto et al., 2022; Saleem et al., 2022). * Hish et al. (2019) and Nagy et al. (2019) represent two separate analyses of the same sample. † Both Kusurkar et al. studies represent two separate analyses of the same sample.

**Table 2:** Psychological Outcomes, Predictors, Protective Factors and Key Findings (n = 24 − excludes intervention studies (detailed in Table 3).

Author/Year	Psychological Outcome	Predictors	Protective Factors	Mediators	Key Findings	Specific/Additional Findings
Ahalli et al. (2022)	Depression, anxiety, job strain	Complementary teaching activity, job strain, poor supervisor relationship, psychiatric history, coexistence of anxiety and depression	Better supervisor relationship; absence of teaching duties (inferred)	Not examined (bivariate analyses only)	34% depressive symptoms, 19% anxiety; poor supervisor relationship and teaching duties as key risk factors	19.3% referred for psychiatric/psychological care; 3.1% suicidal ideation (exclusively male); women—lower decision latitude; 31.8% job strain; participation rate 98%
Alves et al. (2025)	Negative emotions (loneliness, exhaustion, anxiety, depression, despair); positive emotions (satisfaction, relief, joy)	Overemphasis on accountable outputs (publications, rankings); work overload; lack of autonomy; time-management difficulties; work–life imbalance; pressure to publish; weak labour market	Career agency; personal growth; knowledge acquisition; positive relationships with supervisors; passion for research	Tensions between value dimensions (e.g., performativity vs. fulfilment)	Three dimensions of doctoral value: personal/academic/career agency; knowledge outputs; socioeconomic impact; coexist but mutually hindering when one overemphasised	61% negative emotions/mental health problems; health sciences—more intense emotional burden; PhD candidates—career concerns predominant
Bazrafkan et al. (2016)	Stress; anxiety; depression; emotional exhaustion; physical symptoms (headaches, insomnia, panic attacks)	Thesis as major stressor (topic selection, time management, poor writing, lack of resources); non-responsive supervisors; lack of feedback; ineffective supervision; socioeconomic problems; fear of unemployment	Efficient communication with peers/family; finalising thesis (completion as resolution); passion for research	Supervisory quality; socioeconomic pressures	Stress/anxiety/depression in all participants; thesis and supervision as central sources; ineffective supervision as key risk factor	Female students—more emotional distress; coping strategies (music, internet)—temporary relief only; thesis completion as primary coping resolution
den Bakker et al. (2024)	Motivation (autonomous vs. controlled); well-being; imposter feelings; burnout risk	Controlled motivation (PhD as credential for specialty training); fear of failure; poor supervisor fit; competitive peer environment; dependency on supervisor for career outcomes	Autonomous motivation; intrinsic research interest; supportive supervisor; peer support; autonomy at appropriate phase; overcoming challenges as personal growth	Autonomy dosage (phase-dependent); initial motivation type	Six themes influence autonomous and controlled motivation; imposter feelings linked to PhD as proof of competence; positive/negative factors co-occur and change over time	Cognitive dissonance and sunk cost effects sustain controlled motivation when autonomous motivation is depleted; imposter feelings most prominent in early PhD phase
Gao et al. (2025)	Burnout	Social pressure, expressive suppression, advanced year of study, low interest in specialty, difficulties communicating with patients	Cognitive reappraisal, interest in specialty, good health, physical activity	Cognitive reappraisal and expressive suppression between social stress and burnout	Social stress is the strongest predictor of burnout; emotional regulation partially mediates this relationship	Burnout increases with year of study; social stress stronger predictor of burnout than clinical workload
Ghaseminangi et al. (2025)	Emotional fatigue; anxiety; burnout; psychological vulnerability; loss of motivation	Supervisory inconsistency; power imbalance; curriculum misalignment; institutional ambiguity; publication pressure; funding inequity; work–life imbalance	Respectful supervisor communication; peer support and mentoring; transparent institutional procedures	Peer support; supervisory quality	Doctoral challenges interconnected across ecological systems; emotional burden highest during intense research phases; structural constraints amplify difficulties	Female candidates—higher emotional distress; doctoral education governed by two ministries with conflicting requirements, adding stress and confusion for students
Gin et al. (2021)	Depression; low self-esteem; imposter feelings; lack of motivation; difficulty concentrating	Research failures/setbacks; unstructured research environment; negative reinforcement (harsh mentor feedback); unreasonable expectations; social comparison; lack of technical support; social isolation	Teaching (structure, positive reinforcement, passion); collaboration; research passion; emotionally supportive supervisor	Structured tasks in teaching; positive reinforcement from teaching	Research worsened depression; teaching helped depression; depression exclusively negative for research; increased compassion as teachers	Four overarching factors: structure, positive/negative reinforcement, success/failure, social support/isolation; imposter feelings explicitly linked to depression in 58% of sample
Glorieux et al. (2025)	Dropout	Time pressure, teaching assistant role	Supervisor support, passion for research, female gender	/	Dropout is a systemic phenomenon despite long-standing selection; predictors vary across disciplines	Time pressure significant predictor of dropout in Life Sciences & Medicine; female students less likely to drop out
Hish et al. (2019)	Burnout, depression	Academic stress (burnout), family/financial stress (depression)	/	Mastery and supervisor relationship (stress–burnout); mastery (stress–depression)	Different stressors predict different outcomes; mastery is the key mediator in both relationships	Social support not a significant mediator for burnout or depression; correlated only with depression, not burnout
Jachim et al. (2023)	Depression; anxiety; life satisfaction	Poor overall health; female gender; LGBTQ+ identity; food insecurity; lower financial confidence; poorer academic progress (life satisfaction)	Good overall health; financial confidence; social support; satisfaction with mentorship	/	21% depression (2019), 28% (2020), 23% (2021); 36% anxiety (2020), 38% (2021); food insecurity doubled from 11% (2020) to 25% (2021); 40–47% symptomatic students did not seek mental healthcare (“treatment gap”); third-year students showed highest mental health burden	Female and LGBTQ+ students reported higher rates of depression and anxiety across all years
Kusurkar et al. (2021)	Burnout, engagement	Poor sleep quality; frustration of basic psychological needs; work–life imbalance; clinical work environment	Good sleep quality, good work–life balance, autonomous motivation, satisfaction of basic psychological needs	Frustration of basic psychological needs (between work–life imbalance and burnout)	The most important predictors of burnout are poor sleep quality and frustration of basic psychological needs; preventing frustration is more important than ensuring need satisfaction	53% of doctoral students had moderate or high burnout; women had poorer work–life balance; clinical PhD students showed greater autonomy frustration and work-role conflict, not significantly higher burnout
Kusurkar et al. (2022)	Stress and well-being	Research challenges beyond capability, limited resources, lack of recognition, restricted autonomy, poor workplace relationships	Optimal challenges and resources, supportive supervision, autonomy, recognition of work, good workplace relationships	Frustration vs. satisfaction of basic psychological needs (autonomy, competence, relatedness)	Five overarching themes identified (Research is challenging, Resources can be limited, Recognizing the value of work, Experience of autonomy, Relationships are key to success), each containing both stressors and energizers	Relatedness with colleagues most cited energizer; time pressure due to contract expiry most cited stressor; no energizers in Time and Uncertainty categories
Lonka et al. (2019)	Well-being (lack of interest), study satisfaction	Belief in innate writing ability, writing blocks, low knowledge transformation	Knowledge transformation, high writing productivity, constructive feedback	/	Writing beliefs predict satisfaction and lack of interest, but not stress or exhaustion	High stress, exhaustion and anxiety across all profiles
Ma et al. (2024)	Depression, anxiety	Mastery-avoidance goal; performance-avoidance goal	Mastery approach goal	Self-esteem and perceived stress (independently and in chain)	Mastery-avoidance and performance-avoidance goals predict depression and anxiety through chain mediation of self-esteem and perceived stress	22.45% moderate/severe depression, 31.13% moderate/severe anxiety
Mahsood et al. (2025a)	Well-being deficits (mental, physical, emotional); burnout; stress; anxiety; exhaustion; sleep deprivation	Academic overload; inadequate resources/lab infrastructure; administrative delays; financial constraints; publication pressure (high-impact factor journals); supervisory challenges; gender roles and cultural norms	Supervisor and peer support; family and social support; professional networking	/	Well-being multidimensional across five ecological levels; female scholars face double burden (academic + family/cultural responsibilities); policy reforms needed at national and institutional levels	Coursework-heavy PhD structure as source of unnecessary stress; publication costs compound financial burden
Matheka et al. (2020)	Doctoral student success (pace of progress)	Older age, humanities/social sciences	/	/	Motivation and self-efficacy generally do not predict the pace of doctoral study in Kenya; structural factors (age, field of study) more important predictors than psychological ones	Intrinsic motivation predicts faster pace only in medical sciences and among students aged 51+; only 19% of students on track; extrinsic motivation not significant in any subgroup
Nagy et al. (2019)	Burnout; depression; psychiatric diagnoses; dropout ideation	Current psychiatric disorder; functional impairment; dropout ideation; poor employment prospects; poor program climate; poor research training environment quality	Not identified	/	~50% met criteria for at least one current psychiatric disorder; >2/3 reported thoughts of dropping out; burnout associated with dropout ideation, depression and poor program climate; sociodemographic variables did not predict burnout	Anxiety disorders most prevalent current diagnosis (31.9%); 10.1% moderate-to-severe depressive symptoms; 34.4% sought mental health services in past year
Remich et al. (2016)	Gender-based identity threat; stereotype threat; cognitive burden from managing gender presentation	Gender bias (direct and observed); systemic underrepresentation; male-dominated academic culture	Gender identity as source of pride/strength; female role models; supportive partner/family	Gender-explicit strategies mediate persistence	86% acknowledged systemic gender inequities; 100% used at least one gender-explicit strategy; gender biases persist despite perception of improvement	Women often hold contradictory views on gender bias (acknowledge systemic inequities but deny personal experience)
Saleem et al. (2022)	Academic engagement	Stress	Positive emotions, PsyCap	PsyCap partially mediates positive emotions and engagement; stress moderates PsyCap and engagement	Positive emotions increase PsyCap and academic engagement; stress negatively moderates the effect of PsyCap on engagement	PsyCap mediation is partial; positive emotions have a direct effect on engagement independent of PsyCap
Schad et al. (2022)	Depression; anxiety; hazardous alcohol use; drug use; suicidal ideation	Biomedical PhD training; minority race/ethnicity; female gender; LGBQ+ identity	/	/	Biomedical PhD students showed significantly higher depression and anxiety than medical students; racial/ethnic minority students ~1.5× more likely to be depressed/anxious; LGBQ+ students >2× more likely to experience depression and anxiety; suicidal ideation higher in PhD vs. MD students	Medical student mental health improved 2019–2020 while PhD students showed no change; PhD students had markedly higher suicidal ideation in last 12 months in 2020
Souza et al. (2023)	Depression	In final model: anxiety, mania, dissociation; in bivariate analysis also: no partner, low income, professional programme, anger, somatic symptoms, suicidal ideation, sleep disorder, memory, repetitive thinking, personality functioning, substance use	/	/	Anxiety, mania and dissociation are the only significant predictors of depression in the final model; anxiety is the strongest predictor	Depression associated with 11 of 13 DSM-5 domains in bivariate analysis; anxiety, mania and anger showed highest prevalence ratios; 38.5% self-reported sleep disorder”
Tan et al. (2023)	Depression, anxiety	Psychological stress (employment, academic, interpersonal pressure); negative coping style; female gender	Positive coping style, social support	Negative coping style and social support mediate stress and anxiety/depression; social support is the stronger mediator	Stress directly predicts anxiety and depression; negative coping style and social support partially mediate this relationship	21.6% depressive symptoms; 9.4% anxiety; 3rd-year master’s students under greatest employment pressure; 2nd-year students under greatest academic and interpersonal pressure
Tikkanen et al. (2021)	Burnout and research engagement—combined as well-being profiles	Working alone/without a research group	Working in a clinical unit/hospital	/	Four well-being profiles: high engagement–low/moderate burnout, moderate engagement–moderate/high burnout; working in a clinical unit and research group linked to higher engagement, lower burnout risk	Gender, country of origin and full-time/part-time status did not predict profiles; dropout intention dramatically higher in high burnout profile (74.7%) vs. low burnout profile (7.2%)
Chakraverty et al. (2022)	Impostor phenomenon (moderate to intense)	Difficulties forming professional identity; fear of evaluation; minority status and microaggressions; programme phase transitions	Not identified	/	Moderate to intense impostor phenomenon in all participants; four themes connected through ‘othering’; more intense experience in racial/ethnic minority participants	CIPS range 46–96, M = 74.1; one of first studies of impostor phenomenon in MD-PhD population

**Table 3:** Intervention Studies: Outcomes and Study Limitations (n = 3).

Author/Year	Intervention	Intervention Outcome	Study Limitations
Howells et al. (2017)	Gratitude practice − workshop + weekly diary + semi-structured interview	Students: improved personal well-being, reduced stress, increased motivation, resilience and research focus. Supervisors: increased role satisfaction, better communication and feedback quality. Student–supervisor relationship: increased trust, more open communication, increased productivity, reduced power imbalance	Small sample, self-reporting, lacks quantitative validation, socio-cultural differences not systematically examined
Prieto et al. (2022)	Progress-oriented workshops: mental health and productivity education, peer experience sharing, self-tracking and reflection via journaling and dashboard	Significant increase in PsyCap after workshops; initial burnout negatively moderated intervention benefits	Small samples per iteration; no control group or randomisation; author dual role (designer + evaluator); female over-representation; long-term effects not measured
White et al. (2018)	Behavioural self-care intervention integrated into course curriculum; 4 modules (nutrition, mental health, physical activity, social support); SMART goals; bonus points as incentive	Significant improvement in nutrition and physical activity; depressive and anxious days did not significantly increase	No control group; brief mental health measures (single item); ceiling effect; sample included medical residents; possible confounding due to 2016 presidential elections

**Table 4:** Qualitative studies: Main Findings and Links to Review Constructs (n = 8).

Author/Year	Main Findings	Links to Review Constructs
den Bakker et al. (2024)	(1) Initial motivation to start PhD matters; (2) autonomy at the right dose and time; (3) PhD as proof of competence vs. learning trajectory (→ imposter feelings); (4) supervision quality (‘it takes two to tango’); (5) peers can make or break your PhD; (6) strategies to stay/get back on track (active solution-seeking vs. passive acceptance)	Burnout risk (controlled motivation, fear of failure); imposter feelings (explicitly named, linked to PhD as proof of competence); research engagement (autonomous motivation, supervision, peer support)
Ghaseminangi et al. (2025)	Supervisory inconsistency, emotional burden, power imbalance; curriculum misalignment, peer isolation; institutional ambiguity, funding constraints; publication pressure, career uncertainty, gender norms	Addresses burnout (emotional burden theme), engagement barriers (supervisory and structural), and PhD experience in non-Western transitional context; gender disparities in distress relevant to review
Alves et al. (2025)	Three dimensions of doctoral value: (1) personal/academic/career agency; (2) knowledge outputs; (3) socioeconomic impact; dimensions coexist but mutually hindering when one overemphasised	Burnout (emotional burden, anxiety, exhaustion); research engagement barriers (supervisory inconsistency, structural constraints); gender disparities in distress; non-Western transitional context
Gin et al. (2021)	Research worsens depression (failures, unstructured environment, negative reinforcement, isolation); teaching helps depression (structure, positive reinforcement, passion); depression reduces motivation, self-esteem, concentration; increases empathy as teacher	Burnout (depression closely related); imposter feelings (explicitly identified in 58%); research engagement (depression reduces engagement; teaching partially compensates)
Bazrafkan et al. (2016)	(1) Thesis as major stressor (topic selection, time management, poor writing, lack of resources); (2) supervisory relationship (non-responsiveness, lack of feedback); (3) socioeconomic problems (financial strain, fear of unemployment); (4) coping strategies (communication, distraction, thesis completion)	Burnout (thesis and supervisory sources); engagement (thesis completion as coping strategy); supervisory relationship as central moderator
Remich et al. (2016)	Gender-conscious experiences: direct bias, observed bias, systemic inequities; gender-explicit strategies: women’s/sexual health research, gender as source of pride, managing gender presentation, family planning; gender-agnostic strategies: generational change, science as meritocracy	Stereotype threat and cognitive burden of managing gender presentation as barriers to research engagement; gender-explicit strategies (pride, role models, family planning) support persistence toward academic careers
Mahsood et al. (2025a)	(1) Individual: mental/physical/emotional health and work–life balance; (2) Interpersonal: supervisor, peer and family support; (3) Institutional: administrative/financial support and academic resources; (4) Community: professional networking and cultural/gender influences; (5) Policy: national/institutional policy gaps and financial/research policy	Addresses burnout (stress, burnout, emotional exhaustion at individual level), research engagement (academic resources, supervisory support, professional networking), and gender influences on PhD well-being in Pakistani context; policy-level analysis unique among included studies
Kusurkar et al. (2022)	(1) Research is challenging; (2) resources can be limited; (3) recognizing the value of work; (4) experience of autonomy; (5) relationships are key to success—each theme contains both stressors and energizers	Burnout risk (frustration of autonomy, competence, relatedness); well-being and research engagement (satisfaction of same needs); supervisory support and peer relationships as key energizers; time pressure and limited resources as key stressors

**Table 5:** Studies Classified Under Multiple Methodological Categories (n = 5).

Study	Cross-Sectional	Qualitative	Longitudinal	Mixed-Methods	Intervention
Jachim et al. (2023)	√		√		
Howells et al. (2017)		√			√
Prieto et al. (2022)				√	√
Remich et al. (2016)		√	√		
White et al. (2018)			√		√

Note. Five of the 27 included studies were assigned to more than one methodological category based on their reported design; each such study is counted once within each applicable category, which accounts for any apparent discrepancy between the sum of individual category counts and the total number of included studies.

**Table 6:** Summary of MMAT (2018) Quality Appraisal for Included Studies (n = 27).

Study	MMAT Category	S1	S2	Qualitative Criteria (n/5)	Quantitative Criteria (n/5)	Mixed Methods Criteria (n/5)
Ahalli et al. (2022)	4—Quantitative descriptive	Yes	Yes	n/a	5	n/a
Alves et al. (2025)	1—Qualitative	Yes	Yes	5	n/a	n/a
Bazrafkan et al. (2016)	1—Qualitative	Yes	Yes	4	n/a	n/a
den Bakker et al. (2024)	1—Qualitative	Yes	Yes	5	n/a	n/a
Chakraverty et al. (2022)	5—Mixed methods	Yes	Yes	5	2	0
Gao et al. (2025)	4—Quantitative descriptive	Yes	Yes	n/a	5	n/a
Ghaseminangi et al. (2025)	1—Qualitative	Yes	Yes	5	n/a	n/a
Gin et al. (2021)	1—Qualitative	Yes	Yes	5	n/a	n/a
Glorieux et al. (2025)	3—Quantitative non-randomized	Yes	Yes	n/a	3	n/a
Hish et al. (2019)	4—Quantitative descriptive	Yes	Yes	n/a	3	n/a
Howells et al. (2017)	1—Qualitative	Yes	Yes	4	n/a	n/a
Jachim et al. (2023)	4—Quantitative descriptive	Yes	Yes	n/a	3	n/a
Kusurkar et al. (2021)	4—Quantitative descriptive	Yes	Yes	n/a	3	n/a
Kusurkar et al. (2022)	1—Qualitative	Yes	Yes	4	n/a	n/a
Lonka et al. (2019)	4—Quantitative descriptive	Yes	Yes	n/a	3	n/a
Ma et al. (2024)	4—Quantitative descriptive	Yes	Yes	n/a	4	n/a
Mahsood et al. (2025a)	1—Qualitative	Yes	Yes	5	n/a	n/a
Matheka et al. (2020)	4—Quantitative descriptive	Yes	Yes	n/a	3	n/a
Nagy et al. (2019)	4—Quantitative descriptive	Yes	Yes	n/a	3	n/a
Prieto et al. (2022)	5—Mixed methods	Yes	Yes	4	2	3
Remich et al. (2016)	1—Qualitative	Yes	Yes	5	n/a	n/a
Saleem et al. (2022)	4—Quantitative descriptive	Yes	Yes	n/a	4	n/a
Schad et al. (2022)	4—Quantitative descriptive	Yes	Yes	n/a	3	n/a
Souza et al. (2023)	4—Quantitative descriptive	Yes	Yes	n/a	3	n/a
Tan et al. (2023)	4—Quantitative descriptive	Yes	Yes	n/a	5	n/a
Tikkanen et al. (2021)	4—Quantitative descriptive	Yes	Yes	n/a	3	n/a
White et al. (2018)	3—Quantitative non-randomized	Yes	Yes	n/a	2	n/a

S1 = Are there clear research questions? S2 = Do the collected data allow to address the research questions? Consistent with MMAT guidance, criterion ratings were not aggregated into a single overall score; the number of criteria rated “Yes” (out of 5) is reported separately for each applicable component (qualitative, quantitative, and/or mixed methods). For mixed methods studies, the qualitative and quantitative components were each appraised against the criteria of their respective methodological tradition, in addition to the five mixed-methods-specific criteria (MMAT criterion 5.5). n/a—not applicable.

## Data Availability

No new data were created or analyzed in this study. Data sharing is not applicable to this article.

## Abbreviations

The following abbreviations are used in this manuscript:

C-DES	Cross-Country Doctoral Experience Survey
CESD-R	Center for Epidemiologic Studies Depression Scale–Revised
CIPS	Clance Impostor Phenomenon Scale
DASS-21	Depression Anxiety Stress Scale-21
GAD-7	Generalized Anxiety Disorder scale-7
ICD-11	International Classification of Diseases (11th revision)
IS	Impostor syndrome
MBI	Maslach Burnout Inventory
MBI-HSS-MP	Maslach Burnout Inventory–Human Services Survey for Medical Personnel
MBI-SS	Maslach Burnout Inventory–Student Survey
MD-PhD	Doctor of Medicine–Doctor of Philosophy
OR	Odds ratio
PEO	Population, Exposure, Outcome
PHQ-9	Patient Health Questionnaire-9
PRISMA	Preferred Reporting Items for Systematic Reviews and Meta-Analyses
SBI	School Burnout Inventory
SCID-5-PD	Structured Clinical Interview for DSM-5 Personality Disorders
SCID-5-RV	Structured Clinical Interview for DSM-5, Research Version
UWES	Utrecht Work Engagement Scale
UWES-S-9	Utrecht Work Engagement Scale–Student version (9-item)
WoS	Web of Science
